# Severe Left Ventricular Hypertrophy, Small Pericardial Effusion, and Diffuse Late Gadolinium Enhancement by Cardiac Magnetic Resonance Suspecting Cardiac Amyloidosis: Endomyocardial Biopsy Reveals an Unexpected Diagnosis

**DOI:** 10.1155/2016/2461502

**Published:** 2016-05-10

**Authors:** Nina P. Hofmann, Sorin Giusca, Karin Klingel, Peter Nunninger, Grigorios Korosoglou

**Affiliations:** ^1^Department of Cardiology & Vascular Medicine, GRN Hospital Weinheim, 69469 Weinheim, Germany; ^2^Department of Molecular Pathology, University Hospital Tübingen, Tübingen, Germany; ^3^Department of Radiology, GRN Hospital Weinheim, 69469 Weinheim, Germany

## Abstract

Left ventricular (LV) hypertrophy can be related to a multitude of cardiac disorders, such as hypertrophic cardiomyopathy (HCM), cardiac amyloidosis, and hypertensive heart disease. Although the presence of LV hypertrophy is generally associated with poorer cardiac outcomes, the early differentiation between these pathologies is crucial due to the presence of specific treatment options. The diagnostic process with LV hypertrophy requires the integration of clinical evaluation, electrocardiography (ECG), echocardiography, biochemical markers, and if required CMR and endomyocardial biopsy in order to reach the correct diagnosis. Here, we present a case of a patient with severe LV hypertrophy (septal wall thickness of 23 mm, LV mass of 264 g, and LV mass index of 147 g/m^2^), severely impaired longitudinal function, and preserved radial contractility (ejection fraction = 55%), accompanied by small pericardial effusion and diffuse late gadolinium enhancement (LGE) by cardiac magnetic resonance (CMR). Due to the imaging findings, an infiltrative cardiomyopathy, such as cardiac amyloidosis, was suspected. However, amyloid accumulation was excluded by endomyocardial biopsy, which revealed the presence of diffuse myocardial fibrosis in an advanced hypertensive heart disease.

## 1. Introduction

Increased left ventricular (LV) wall thickness, referred to as LV hypertrophy, is a powerful predictor of cardiovascular morbidity and mortality [1]. LV hypertrophy can be related to a multitude of cardiac or systemic diseases such as hypertrophic cardiomyopathy (HCM), cardiac amyloidosis, Anderson-Fabry disease, and hypertensive heart disease [2–4]. The clinical presentation of all of these disorders is highly variable from being asymptomatic to sudden death, whereas they usually lead to congestive heart failure in their advanced stages.

Amyloidosis is characterized by the extracellular deposition of amyloid, as a result of misfolding of a precursor protein, the most common of which are light chains, transthyretin, and serum amyloid A [5]. Cardiac involvement is common and represents an adverse predictor of outcome with amyloidosis [4]. HCM, on the other hand, is caused by mutation of sarcomeric genes and is associated with concentric LV hypertrophy [3]. In some patients, HCM progresses to congestive heart failure with poor clinical outcome [6]. In addition, arterial hypertension is the most common cause of LV hypertrophy due to pressure overload, which can also progress to hypertensive heart disease associated with heart failure with or without preserved LV ejection fraction [2]. Due to advances in the antihypertensive treatment, the burden of hypertensive heart diseases decreased during the last decades [7].

In clinical routine, the diagnostic workup of patients with LV hypertrophy is important due to specific therapeutic strategies aiming at the different disease etiologies. Here, we present a case where the presence of cardiac amyloidosis was suspected based on cardiac imaging findings. However, endomyocardial biopsy revealed an unexpected diagnosis.

## 2. Case Presentation

**Figure 1:**
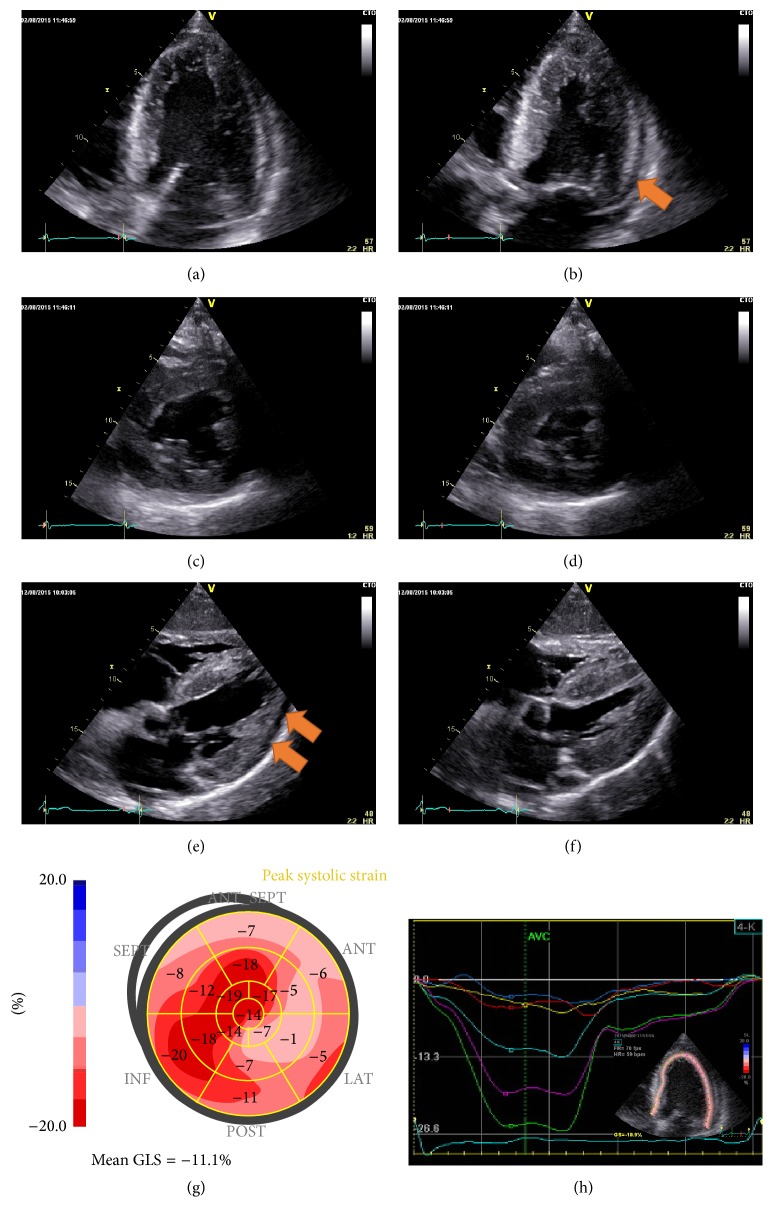
Echocardiographic images revealing severe left ventricular hypertrophy ((a)–(f)). Reduced longitudinal function was noticed in the presence of preserved radial deformation and ejection fraction ((g)-(h)). Minimal pericardial effusion was noticed (orange arrows in (b) and (e)).

A 56-year-old male patient was referred to our department due to exertional dyspnea and gradual decrease of his exercise tolerance within the last 6 months. The patient had no history of cardiac diseases and no history of arterial hypertension, hyperlipidemia, and diabetes mellitus. Physical examination revealed normal heart sounds, no murmurs, and mild peripheral edema with clear lung fields. Blood pressure was elevated at 180/90 mmHg with normal pulse of 58 bpm. An ECG suggested a sinus rhythm without signs of myocardial ischemia or low QRS voltage but negative T-waves in V4–V6. Echocardiography, on the other hand, revealed the presence of severe LV hypertrophy (Figures 1(a)–1(f)) with septal wall thickness of 20 mm, preserved radial wall motion, and LV ejection fraction but strongly diminished longitudinal function (Figures 1(g) and 1(h)) (mitral annular plane systolic excursion (MAPSE) = 7 mm; mean longitudinal strain = −11.1%; ejection fraction = 55%). In addition, granular sparkling of the myocardium was observed and small pericardial effusion was noticed, without hemodynamic relevance (orange arrows in Figures 1(b) and 1(e)). High-sensitive troponin T (hsTnT) was elevated (32 pg/mL), whereas renal function and C-reactive protein were normal. In addition, light chains and protein in urine were within the normal range (Table 1).

**Figure 2:**
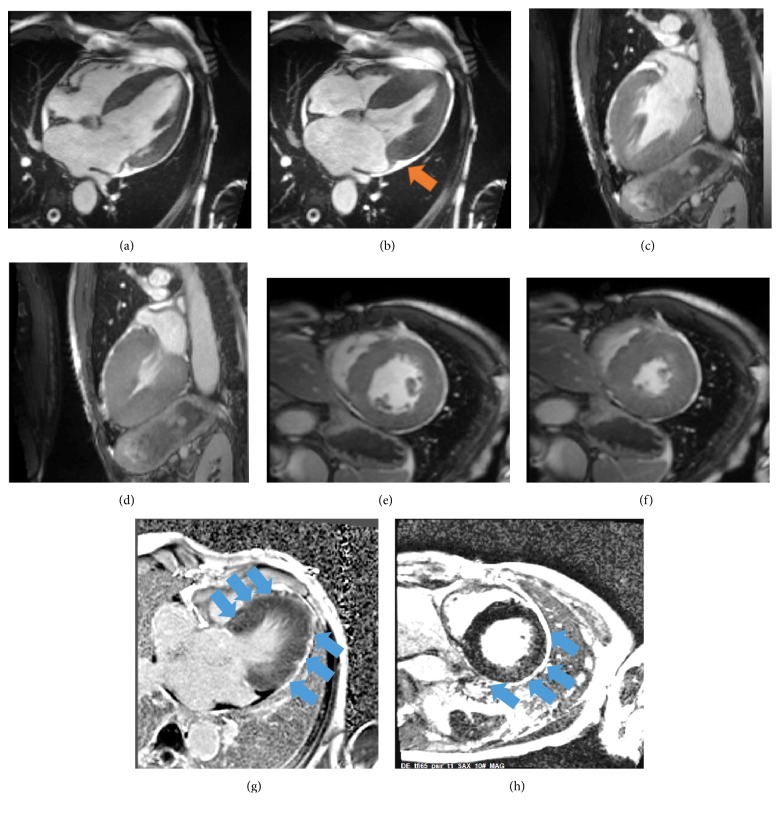
Cardiac magnetic resonance imaging confirmed severe left ventricular hypertrophy and minimal pericardial effusion ((a)–(f), orange arrow in (b)). Late gadolinium enhancement was suspected in corresponding 4-chamber and short axis view images ((g)-(h)).

**Figure 3:**
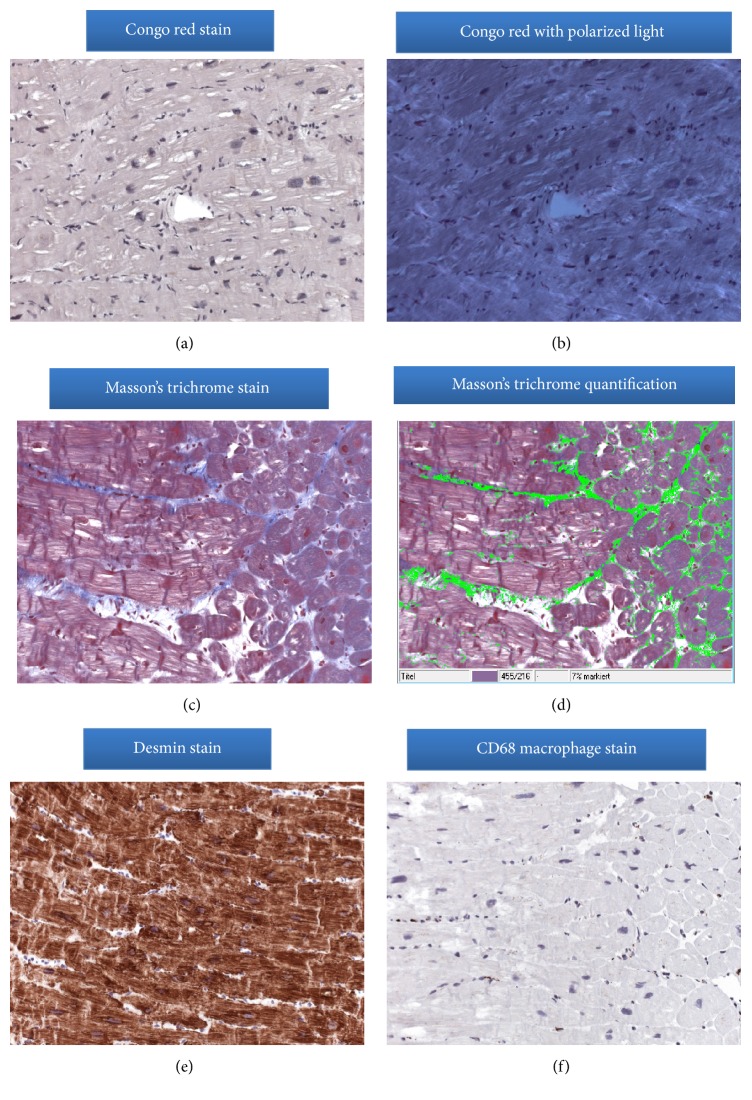
Congo red stain excluded the presence of amyloid in the heart ((a)-(b)), while Masson's trichrome stain showed diffuse hypertrophy of myocytes and an interstitial fibrosis with the myocardium (blue areas (c), area of fibrosis of 7% by quantification analysis in (d)), indicating the presence of hypertensive heart disease. In addition, immunohistochemical stain for desmin and CD68 suggested the absence of myocardial texture disorders and macrophage infiltration ((e)-(f)).

Because echocardiographic findings were indicative of a myocardial infiltrative disease, cardiac magnetic resonance (CMR) was subsequently performed. CMR confirmed the presence of severe LV hypertrophy (septal wall thickness: 23 mm; lateral wall thickness: 18 mm; LV mass: 264 g; and LV mass index: 147 g/m^2^), small pericardial effusion, and impaired longitudinal wall motion with an ejection fraction of 54% (Figures 2(a)–2(f)). In addition, late gadolinium enhancement exhibited a diffuse LGE pattern in the 4-chamber view and patchy LGE pattern in the inferior-lateral wall, suspecting cardiac amyloidosis (Figures 2(g) and 2(h)). Based on all the echocardiographic and CMR imaging findings and due to suspected cardiac amyloidosis, the patient was scheduled for cardiac catheterization and endomyocardial biopsy. Coronary angiography excluded the presence of significant coronary artery disease and several biopsies could be harvested from the left ventricle. From paraffin-embedded cardiac tissue, routine cardiopathologic staining and immunohistochemistry were performed. Congo red stain excluded the presence of amyloid in the heart. In addition, no apple-green birefringence was observed in the heart tissue of our patient under polarized light in Figures 3(a) and 3(b). Masson's trichrome stain, on the other hand, demonstrated the presence of cardiomyocyte hypertrophy and a diffuse interstitial fibrosis including microfoci of collagen scars (blue areas in Figure 3(c)). Quantification analysis as obtained by the programme Quantuepatho showed an area of fibrosis of 7% in our patient (Figure 3(d)) [8]. This percentage is higher compared to normal heart tissue, which contains very low amounts of fibrous perimysial collagen and less than 3% fibrosis [9]. In addition, immunohistochemical stains for desmin and CD68 stains suggested the absence of myocardial texture disorders and macrophage infiltration (Figures 3(a)–3(f)).

The patient was put on antihypertensive medication with 190 mg metoprolol, 10 mg ramipril, 250 mg clonidine retard, 50 mg dihydralazine, 10 mg amlodipine, 8 mg doxazosin, and 50 mg triamterene with 25 mg hydrochlorothiazide.

Ambulatory follow-up in 3 months revealed office blood pressure of 130/70 mmHg and recovery of all symptoms including exertional dyspnea. Echocardiography showed similar findings of LV hypertrophy (septal wall thickness of 20 mm) comparable to the initial findings.

## 3. Discussion

The morphological findings and clinical features of LV hypertrophy may be similar to disorders, such as HCM, amyloidosis, and hypertensive heart disease, so misdiagnosis can frequently occur. Our case demonstrates the presence of severe LV hypertrophy, small pericardial effusion, and late gadolinium enhancement by CMR, so infiltrative disease, such as cardiac amyloidosis, was suspected. However, myocardial biopsy revealed the presence of hypertensive heart disease, which could be effectively treated using a combination of antihypertensive drug therapies.

Clinical presentation of patients with LV hypertrophy may differ between absence of clinical signs upon presentation and life-threatening arrhythmias or symptomatic congestive heart failure. Although the 12-lead ECG may be helpful for the identification of HCM [10], such patients might in some cases present with normal ECG. In addition, patients with amyloidosis may exhibit low total 12-lead QRS voltage despite the presence of LV hypertrophy [11].

To date, several imaging methods are available for the diagnostic workup of patients with myocardial hypertrophy. Echocardiography represents the first-line imaging modality for the evaluation of LV hypertrophy, allowing the assessment of LV wall thickness, end-diastolic and end-systolic volumes, and LV mass. In addition, the assessment of diastolic function, myocardial strain, and strain rate by echocardiography can add incremental value for the differentiation between cardiac amyloidosis and other myocardial disorders causing LV hypertrophy [12, 13]. Despite its high practicability, however, echocardiography is an operator dependent technique, which is limited by the echogenic windows of the patients.

CMR, on the other hand, exhibits higher spatial resolution than echocardiography, and its versatility allows for the assessment of ventricular thickness and mass, myocardial function (ejection fraction), perfusion, and if required myocardial deformation and viability (scar, fibrosis) by LGE within a single examination and without radiation exposure for the patients [14].

A number of previous studies investigated the ability of CMR to differentiate patients with LV hypertrophy due to HCM, amyloidosis, and hypertensive heart disease. Thus, Sipola et al. [15] suggested that a maximal wall thickness of ≥17 mm could precisely differentiate patients with D175N mutation related HCM from those with hypertensive heart disease. However, in our case, this criterion would not be useful, because our patient exhibited a septal wall thickness of over 20 mm and a maximal wall thickness of 23 mm.

The presence of LGE with hypertensive heart disease, on the other hand, is rated differently in the current literature. Thus, LGE was seen in 14 of 43 patients with HCM but in none of 39 patients with hypertensive heart disease [16]. In the same line, diffuse LGE was observed in 20 of 30 patients with cardiac amyloidosis, but in none of 16 patients with hypertensive heart disease [17]. In a recent European CMR study, on the other hand, 13 of 26 patients with hypertensive heart disease exhibited nonischemic, patchy LGE, predominantly in septal and inferior-lateral myocardial segments [18]. In the same line, another recent study demonstrated patchy (*n* = 4) and midwall (*n* = 2) nonischemic LGE in 6 of 11 patients with hypertensive heart disease, predominantly in septal and inferior regions [19]. Conversely, in our patient, diffuse LGE was suspected in the 4-chamber view and patchy LGE in the inferior and lateral LV wall. LGE is very common in patients with cardiac amyloidosis and represents interstitial expansion from amyloid deposition. Typical findings in cardiac amyloidosis include low T1 difference between the myocardium and the LV cavity and circumferential subendocardial or transmural LGE [20–23]. In our case, the LGE pattern was not typical for amyloidosis. We therefore decided to proceed with myocardial biopsy.

With hypertensive heart disease, on the other hand, left ventricular hypertrophy results from chronic pressure overload due to systemic hypertension. In the absence of antihypertensive treatment, diastolic dysfunction occurs, which may lead to heart failure symptoms. During later stages of the disease, subendocardial interstitial fibrosis is increasing, compromising myocardial contractility and ultimately causing systolic LV dysfunction [24, 25]. Despite advances in the antihypertensive medications, the clinical burden of hypertensive heart disease is nonnegligible, and in some cases heart failure with preserved ejection function is present due to unchecked arterial hypertension [25]. Due to the increased rate of cardiac events with hypertensive heart disease, the early diagnosis and treatment of this disorder are crucial.

Finally, from a technical point of view, T1 mapping may have further helped in differentiating between amyloidosis, hypertrophic cardiomyopathy, and hypertensive heart disease in our case but was unfortunately not yet implemented with our scanner at the time of the study [20, 26].

In conclusion, the diagnostic process with LV hypertrophy requires the integration of clinical evaluation, electrocardiography, echocardiography, and if required CMR, biochemical and serological markers, and endomyocardial biopsy. Although the latest improvements with noninvasive diagnostic techniques have reduced the need for biopsy, in our case, endomyocardial biopsy excluded cardiac amyloidosis, as suspected by CMR, and revealed the presence of hypertensive heart disease.

## Figures and Tables

**Table 1:** Serum and urine laboratory findings.

High-sensitive troponin T	31.8 pg/mL
Serum creatinine	1.28 mg/dL
eGFR (estimated glomerular filtration rate)	>60 mL/min *∗* 1.73 m^2^
C-reactive protein (CRP)	0.8 mg/L
Free light chains in urine	41 mg/L
Free Lambda light chains in urine	<5.3 mg/L
Proteins in urine	97 mg/L
Proteins in urine	195 mg/24 h
